# Individualized markers optimize class prediction of microarray data

**DOI:** 10.1186/1471-2105-7-345

**Published:** 2006-07-14

**Authors:** Pavlos Pavlidis, Panayiota Poirazi

**Affiliations:** 1 Institute of Molecular Biology and Biotechnology (IMBB), Foundation for Research and Technology-Hellas (FORTH), Vassilika Vouton PO Box 1385, GR-71110, Heraklion, Crete, Greece; 2 Department of Biology, University of Crete, PO Box 2208, GR-71409, Heraklion, Crete, Greece

## Abstract

**Background:**

Identification of molecular markers for the classification of microarray data is a challenging task. Despite the evident dissimilarity in various characteristics of biological samples belonging to the same category, most of the marker – selection and classification methods do not consider this variability. In general, feature selection methods aim at identifying a common set of genes whose combined expression profiles can accurately predict the category of *all *samples. Here, we argue that this simplified approach is often unable to capture the complexity of a disease phenotype and we propose an alternative method that takes into account the individuality of each patient-sample.

**Results:**

Instead of using the same features for the classification of *all *samples, the proposed technique starts by creating a pool of informative gene-features. For *each *sample, the method selects a subset of these features whose expression profiles are most likely to accurately predict the sample's category. Different subsets are utilized for different samples and the outcomes are combined in a hierarchical framework for the classification of all samples. Moreover, this approach can innately identify subgroups of samples within a given class which share common feature sets thus highlighting the effect of individuality on gene expression.

**Conclusion:**

In addition to high classification accuracy, the proposed method offers a more individualized approach for the identification of biological markers, which may help in better understanding the molecular background of a disease and emphasize the need for more flexible medical interventions.

## Background

The advent of microarray technology along with the exponential accumulation of biological data have recently led to a massive search for new knowledge that can be used to improve our quality of life, focusing mainly on the alleviation of health problems [1-10]. Identification of molecular markers for various diseases has become a major issue in such tasks [11-13]. Molecular markers are genes that can be used to:

1. discriminate between different disease types

2. predict the outcome of a disease

3. detect sub-categories or states of a disease

4. pin down independent and possibly unknown processes which are involved in the generation or the progression of a disease.

Several marker (or feature) selection methods have been used in gene expression studies utilizing microarray technology. Among these, filter methods in which the selection is independent from the optimization criteria of the classifier are most frequently used. Such methods have the advantage of being cost-effective and easy to implement which make them very attractive for microarray data experiments where the set of features is in the order of thousands. Frequently used filter methods include the two-sample t-test [14-22] Signal-to-Noise [23], TNoM [24], ICED [25] and the z-test [26] just to name a few. Wrapper methods on the other hand use similar criteria as the classifier in order to select optimal features thus maximizing classification capacity. Recursive Feature Elimination is an example of a wrapper method used on microarray data [27]. A recent study [28] comparing the performance of filter vs. wrapper methods on microarray data showed that the latter achieve higher performance than the former but the improvement in performance is accompanied by a considerable cost in computational complexity. While a number of other feature selection methods have been used in microarray data, only the aforementioned filter methods are discussed as they are more relevant to the present work. All of these methods have a number of shortcomings that are particularly important when applied to microarray data. For example, a basic assumption of the t-test and Signal-to-Noise methods is that data follow a normal distribution, a postulation which is not always valid for microarray experiments. In fact, a recent publication [29] showed that a yeast gene expression dataset is better modeled by an alpha distribution (*a *= 1.3). The main difference between Signal-to-Noise and t-test is that the former gives a larger penalty to genes with high expression variance in both -as opposed to just one- classes. However, this kind of expression variability might be important for biological samples, where only a given condition may influence the expression of certain marker genes. The main drawback of these methods is that they both assume a global behavior of a marker gene across all samples of the same class, which is an oversimplified assumption for biological samples.

TNoM [24] is a non-parametric test in which an expression threshold is estimated for each gene and used to assign samples in one or the other class so that the number of prediction errors is minimized. Genes that make the smallest number of misclassifications on a training set are selected as markers. Similar to Signal-to-Noise and t-test, this supervised feature selection method assumes a single expression threshold in selected genes. The ICED (Independently Consistent Expression Discriminator) method introduced by [25] effectively bypasses the normality assumption which is required for t-test-like methods. The method searches for genes which are consistently expressed near a single threshold level in one class and far from this level in the other class. This property adds flexibility by taking into account inherent genetic variation and environmental influence. Finally, Recursive Feature Elimination (RFE) [30] is based on finding features for Support Vector Machines that minimize bounds on the leave-one-out cross validation error. The method uses exhaustive supervised search for the identification of marker genes without making any assumptions about their statistical properties, thus suffering only from time and complexity limitations. Almost all of the aforementioned techniques assume that each dataset contains a number of genes that serve as independent discriminators for disease categories, using a single expression threshold. Specifically, a sample belongs to one category if the expression of a given gene is above threshold and/or to a second category if its expression is below this threshold. However, this may not always be the case, particularly in gene expression data associated with complicated biological processes. The etiology of complex biological events, such as the progression of healthy to cancerous cells, or the response of cancerous tissue to a specific treatment can vary considerably among different patients. This stems from the fact that a large number of molecular processes are implicated in the pathogenesis of cancer, including changes in:

1. signal transduction

2. protein degradation and stability

3. gene regulation

4. immortalization and senescence

5. differentiation

6. cell cycle/checkpoints

7. chemical and radiation- induced mutagenesis

8. metabolism and

9. the stress response (NIH-CE)

The order and magnitude of the change in these processes is highly probable to differ, even among samples of the same cancer type. This variability introduces an important complication: assuming that some/all of the above processes are induced by (or induce) an alteration in the gene expression levels, then samples with different gene expression signatures can develop the same pathogenicity. As a result, identifying a single set of gene markers, capable of accurately characterizing all samples becomes nearly impossible. On the other hand, one could argue that static expression patterns provided by microarray experiments are far from capturing this type of biological complexity. Therefore, searching for a common set of feature genes could be the only way to ensure robustness of classification as well as biological relevancy of selected features. However, if gene expression variations among two or more large groups of patients are due to consistent upstream effects on the same gene(s), as for example the activation of different converging pathways or the recruitment of different transcription factors, such changes could be reflected in static microarray experiments and may worth being investigated further. Our goal in this work is to develop a method that utilizes gene expression information to characterize the status/category of each sample while taking into account its individuality, hoping that this approach will provide a better understanding of the disease under study. Towards this target, we take into consideration two biologically significant forms of variability in the data:

1. Samples belonging to the same class may have considerably different gene expression profiles. This variability is two-fold. First, each sample maybe best characterized by a (partially or entirely) different set of marker genes. Second, each sample maybe best characterized by a different expression range of a common set of gene features. To address this kind of variability, we construct a pool of genes, hereby termed "informative genes", each of which carries important information with respect to the categorization of some -but not necessarily all- samples. Each informative gene comprises of one or more "Consistent Expression Regions" (CERs) that accurately predict the category of certain samples (see Methods).

2. Variability among samples of the same class may indicate the existence of unknown subgroups that should be treated separately. The proposed method is particularly suitable for this kind of variability, as it has an innate property to identify subgroups based on their characterization by a common subset of genes or gene expression regions.

## Results and discussion

The proposed method was applied to several publicly available microarray datasets (most of which can be found at [31]). A comparison between the classification performance of this method and that achieved by previously used techniques is shown in Table 1. As evident from the results, our method outperforms (in 3/6 datasets) or matches (in 3/6 datasets) the performance of all other referenced methods. However, it should be stressed that achieving high classification performance was not the main goal of this work. Similar accuracy on the same datasets has also been reported in several recent publications [32-38]. Perhaps the most important contribution of this work is that, in addition to high classification accuracy, it offers a method for identifying genes of high discrimination value that are most likely to be missed by traditional feature selection techniques due to their complex expression profiles (see Methods). As shown in Table 1 (last column), the number of informative genes that were also identified by the reference publications is quite high in datasets where the majority of the selected genes had simple step-like profiles (1^*st *^order genes). However, this overlap is significantly smaller in cases containing many higher-order genes, such as the CNS dataset, since those features could not be detected in the reference publication (for respective lists of overlapping genes see [Additional files 2–6]). Higher-order genes may be particularly important for understanding the molecular basis of complex diseases as they can naturally divide samples of the same class into distinct sub-groups thus highlighting the effects of individuality on gene expression patterns. Cases of particular interest that exploit the advantages of our method are discussed next, while information about additional datasets used can be found in [Additional file 1].

### AML vs. ALL leukemia results

**Figure 2:**
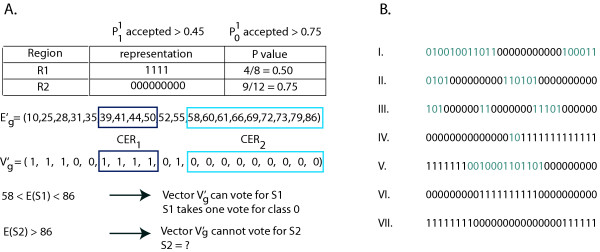
A. Procedure for detection of informative genes and class prediction. The ranked expression profile of gene g (E'g) corresponds to a labeling vector (V'g) which contains two regions that group together a significant number of class 1 and class 0 samples (50% and 75%). These regions map on two CERs: [39-50] and [58-86], respectively. The gene can thus be used for the classification of a new sample S1 in which its expression lies between the respective thresholds of CER2, but not for a sample S2. B. Examples of labeling vectors -that correspond to informative genes- identified by our method. Vectors V and VI are of particular biological interest as they divide samples of class 0 and 1 respectively, in two distinct sub-groups.

The AML/ALL leukemia data set [23,39] was used to identify molecular markers that differentiate between AML and ALL samples. The set contains 47 AML and 25 ALL samples, divided into a training (27 ALL, 11 AML) and a test set (20 ALL, 14 AML). 15 AML samples were derived from patients treated with an anthracycline-cytarabine regimen and the long-term clinical follow-up was available. 8 of these patients failed to achieve remission after induction of chemotherapy, while the rest remained in remission for 46 to 84 months. Furthermore, 9 AML samples were derived from T-cells and the remaining 38 from B-cells. For a significance threshold *p*_*s *_= 1%, a total of 345 genes (250, 60, 15 and 20 1^*st*^, 2^*nd*^, 3^*rd *^and 4^*th *^order, respectively) were selected to form the aggregate classifier (see Methods) and a performance of 33/34 correct classification was achieved. A graphical illustration of this classification is shown in Figure 2 of [Additional file 1]. In addition to class prediction, the method was used to search for sample sub-groups within the two main class categories as discussed in the following sections.

#### Identification of AML sub-groups: "Failure" vs. "Success" discrimination

The probability of success is a crucial factor in medical treatment of leukemia, since many patients respond differentially to available treatments. The method was used to seek for molecular signatures that may provide an explanation for this phenomenon. Towards this target, the method was re-trained to discriminate between ALL and AML classes using all AML samples providing information about the failure or success of treatment (8 "Failure" and 7 "Success") along with 27 ALL samples. Sub-groups for each class were constructed using *tight *sets of marker genes, as described in the Methods section. Among the resulting AML sub-groups, one was able to distinguish "Failure" from "Success" cases, with a single misclassification, utilizing only seven genes as shown in Figure 4. The probability of this discrimination being randomly achieved by the specific set of genes was very low (*p *= 7 × 10^-5^). Detailed information about the respective genes is included in Table 2 of [Additional file 1].

#### Identification of ALL sub-groups: B-cell vs. T-cell sample discrimination

The type of lymphocytes affected (B-cell *vs*. T-cell) provide a sub-classification of ALL samples. The method was used to search for gene markers specific to the two main classes that may additionally explain this differentiation. Training and test sets were merged to form a dataset comprising all 72 samples and the method was re-trained to discriminate between ALL and AML samples. Contrarily to the "Failure" *vs*. "Success" discrimination, none of the *tight *set of genes was able to distinguish B-cell from T-cell samples within the ALL class. However, the usage of all higher-order genes revealed the existence of a single branch within the dendrogram which contains all T-cell samples (Figure 5). Detailed information about the genes that support this grouping is provided in Table 3 of [Additional file 1].

### ALL/MLL/AML leukemia results

The ALL/MLL/AML dataset [40] consists of 72 samples divided in a training (20 ALL, 17 MLL and 20 AML) and a test set (4 ALL, 3 MLL and 8 AML). According to [40], lymphoblastic leukemias with MLL translocations can clearly be separated from conventional acute lymphoblastic (ALL) and acute myelogenous leukemias (AML) suggesting that lymphoblastic leukemias with MLL translocations constitute a distinct disease, denoted as MLL. Using Leave-One-Out Cross Validation (*LOOCV*), our method correctly assigned 56/57 samples to their respective classes. To investigate the possibility of MLL samples forming a distinct category, three pair-wise comparisons in which two of the groups were merged in one class were performed. For the MLL *vs*. ALL-AML comparison, a total of 1451 genes (539, 336, 408 and 168 1^*st*^, 2^*nd*^, 3^*rd *^and 4^*th *^order, respectively) were selected to form the aggregate classifier. For the ALL *vs*. MLL-AML comparison, a total of 4809 genes (2390, 1498, 597 and 324 1^*st*^, 2^*nd*^, 3^*rd *^and 4^*th *^order, respectively) were selected, while for the AML *vs*. MLL-ALL comparison, a total of 7930 genes (3625, 2756, 1113 and 436 1^*st*^, 2^*nd*^, 3^*rd *^and 4^*th *^order, respectively) were selected. The significance threshold for all three comparisons was *p*_*s *_= 1%. All resulting dendrograms assigned MLL samples in a distinct category, which appears more similar to the ALL than the AML class (see Figure 6). This is in agreement with a previous study that used Principal Component Analysis to show that cases with MLL chimeric fusion genes segregate according to their lineage [41]. The expression profile for six of the most important genes that are differentially expressed between the three classes is shown in Figure 6, while more detailed information can be found in Table 5 of [Additional file 1]. Their expression is consistently low in AML samples, high in ALL samples and intermediate in MLL samples.

### Breast cancer results

The breast cancer data set [42] was used to identify molecular markers that differentiate between *ER*^+ ^and *ER*^- ^samples. The data set contains 25 *ER*^+ ^and 24 *ER*^- ^samples, divided into a training (20 *ER*^+^, 20 *ER*^-^) and a test set (5 *ER*^+^, 4 *ER*^-^). For a significance threshold *p*_*s *_= 1%, a total of 508 genes (334, 153, 16 and 5 1^*st*^, 2^*nd*^, 3^*rd *^and 4^*th *^order, respectively) were selected to form the classifier. According to [42] tumor samples 14, 31, 33 were initially classified as *ER*^+ ^by IHC but they were later termed as *ER*^- ^by immunoblotting (hereby marked as *ER*^-*^) whereas samples 45 and 46 were determined as *ER*^- ^by IHC and *ER*^+ ^by immunoblotting (hereby marked as *ER*^+**^). Our results, illustrated in Figure 7, are consistent with the results of [42] except for the classification of sample 14. The expression profile of sample 14 yields an uncertain prediction according to [42] while our method assigns this sample to the *ER*^+ ^class.

### Central Nervous System (CNS) results

The Central Nervous System data set [43] contains 60 biopsy samples taken from patients with various tumor types including medulloblastomas, primitive neuroectodermal tumours (PNETs), atypical teratoid/rhabdoid tumours (AT/RTs) and malignant gliomas. Samples were obtained before the patients received treatment, accompanied with clinical follow-up. Survivors are patients who are alive after treatment, while failures are those who succumbed to their disease. For a significance percentage *p*_*s *_= 2%, a total number of 443 genes (170, 82, 51 and 140, 1^*st*^, 2^*nd*^, 3^*rd *^and 4^*th *^order, respectively) were selected to discriminate between poor *vs*. good treatment outcome. When only first order genes were used in the classifier, the method achieved a performance of 44/60 correct classification. Notice that first order genes roughly resemble genes that are selected by Signal-to-Noise or t-test as done in the original publication, thus explaining the similar classification accuracy (47/60). However, using only higher order genes (*n *> 1) resulted in a significant improvement in discrimination accuracy with 55/60 correct class assignments. This improvement maybe be due to the complexity of the particular tumors that belong to at least four different categories, which can only be captured by more complex (higher order) gene features. Figure 6 in [Additional file 1] provides some supporting evidence for this hypothesis. As evident in the figure, the fraction of higher order genes among all selected features is consistently larger in the CNS as opposed to two other datasets (ALL/AML and Breast Cancer) in which the content of 1^*st *^order genes is much bigger. The statistically significant presence of higher order genes in the CNS data along with the improved classification capacity achieved with these features suggests an important discriminatory and perhaps biological role. A list of six representative higher order genes selected in the CNS dataset is included in Table 4 of [Additional file 1].

## Conclusion

Given a set of tissue-specific microarray experiments performed under different conditions, this work presents a new method for identifying genes that can explain or get affected by these conditions. Such informative genes are shown not only to accurately discriminate between different disease types or stages but also reveal the existence of known or new sub-groupings within a main category and pinpoint molecular mechanisms that are likely to support these groupings.

Unlike existing filter feature selection techniques, this method applies no restrictions to the mean expression values of informative genes between the different classes. Informative genes are identified according to the existence of at least one well defined expression region that corresponds to a significant number of samples of the same class. The underlying assumption is that if the expression of a gene in a significant number of same-class samples ranges within a given interval, then this expression interval may constitute a marker-feature for this sub-group of samples. Moreover, the use of multiple expression regions as distinct classifiers for different sub-groups of samples is based on the hypothesis that a gene does not necessarily need to be down-regulated (or up-regulated) in one class relative to the other in order to be informative. It is possible that a gene contains more than one expression region characteristic of the same class and one or no characteristic region for another class. Such an example is gene KIAA0016 that codes a mitochondrial import receptor (TOMM20) [44], whose expression profile in AML *vs*. ALL samples reveals that both the lower and the higher expression values are characteristic of ALL samples while most of the intermediate values are characteristic of AML samples (see Figure 5 in [Additional file 1]). Although not directly linked to cancer, this receptor has been shown to interact with bcl-2, a central anti-apoptotic protein whose expression is high in both AML and ALL patients, possibly allowing its insertion to the mitochondrial outer membrane [45,46]. Interestingly, the expression of bcl-2 serves as a prognostic marker for remission outcome and long-term survival in AML [47] but not ALL patients where results are controversial [48,49]. Our findings open up the possibility of a TOMM20 involvement, through the bcl-2 interaction, in the differential resistance to chemotherapy evident in AML *vs*. ALL patients. Note that such a gene, although of high discrimination quality as well as biological relevancy, would be missed by most standard statistical feature selection techniques since its mean expression value in each class is approximately the same. Another interesting example is gene SMARCA4 (see Table 4 and Figure 7 in [Additional file 1]) which is found by our method to have a 3-step-like expression profile with low expression values in AML samples, intermediate expression values in MLL samples and higher expression values in ALL samples. This gene was previously shown to be differentially expressed in MLL *vs*. ALL samples [40] as well as between AML and ALL samples [23] but its heterogeneous expression profile and discrimination capacity across all three categories could not be captured in those studies.

The utilization of higher order (multiple-region) genes often results in a significant improvement in discrimination performance. A nice example is the classification of Central Nervous System samples into two classes representing poor *vs*. good treatment outcome, where the utilization of higher order genes alone results in significantly higher accuracy compared to the use of first order genes. Note that first order (single-region) genes are similar to those detected by Signal-to-Noise, t-test or ICED methods as they often have a single-threshold for classification. It is likely however, that treatment outcome in these patients depends heavily on genes with a more complex expression pattern that differentially characterizes the heterogeneous group of CNS tumors used in this study. A comparison between AML/ALL Leukemia, Breast Cancer, and Central Nervous System datasets -all of which are performed using the same microarray chips – reveals several interesting differences in the number and order of selected genes. First order genes comprise nearly 70% of the total number of selected genes in both Breast Cancer and AML/ALL Leukemia datasets, but less than ~40% in the Central Nervous System dataset. On the contrary, more higher-order (4^*th *^and 3^*rd*^) genes are selected in the Central Nervous System dataset as compared to the other two, supporting the hypothesis that treatment outcome for CNS tumor patients is characterized by complex gene expression patterns (see Figure 6 in [Additional file 1]). Moreover, a number of higher order genes selected by our method have been associated with CNS tumors and treatment outcome. Interesting examples include the gene encoding for CD70/CD27 ligand, the antiapoptotic gene seladin-1, the gene coding for the interleukin-1 receptor (IL1R1) and the gene coding for the Ser/Thr protein kinase CDK5 (see Table 4 in [Additional file 1]). CD70 is a member of the Tumor Necrosis Factor family which is highly expressed in human brain tumors [50] and was recently shown to play an immune stimulatory role -preventing tumor growth in vivo- that encourages its application in tumor immunotherapy [51]. The interleukin-1 receptor (IL1R1) is a membrane protein which is variably expressed in different brain tumors [52] and has also been suggested to play a role in brain immunotherapy of astrocytomas [53]. The antiapoptotic gene seladin-1, which is implicated in Alzheimer's disease and cholesterol metabolism, was also found to integrate cellular response to oncogenic and oxidative stress [54]. This gene was recently found to be downregulated in adrenocortical adenomas and carcinomas [55] while its differential expression in pituitary adenomas has been suggested to associate with a different apoptotic response to somatostatin analogs [56]. Cyclin dependent kinase 5 (CdK5) is a proline-direct protein kinase that is most active in the CNS and has been implicated in certain neurodegenerative diseases. It was recently shown to facilitate the progression of apoptosis by regulating the activity of the tumor suppressor protein p53 [57], the expression of which has been associated with poor prognosis in primary CNS diffuse large B-cell lymphoma [58]. In addition, overexpression of both p53 and bcl-2 proteins has been associated with ominous prognosis in pediatric glioblastoma multiforme tumours [59]. Taken together, these findings suggest that genes with heterogeneous expression detected by our method are not simply the result of technical or biological irrelevant variation but they can have an important biological role.

**Figure 3:**
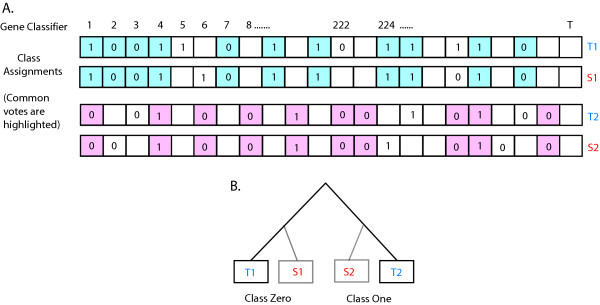
Procedure for building a dendrogram. Using the distance metric shown in equation 1, we determine a similarity score between two samples that reflects the number of common votes provided by the same informative genes. A. Based on this score, samples T1 and T2 in the training set form a bifurcation note representing their respective classes. B. Unseen samples S1 and S2 are placed on this node according to their similarity with T1 and T2.

**Figure 4:**
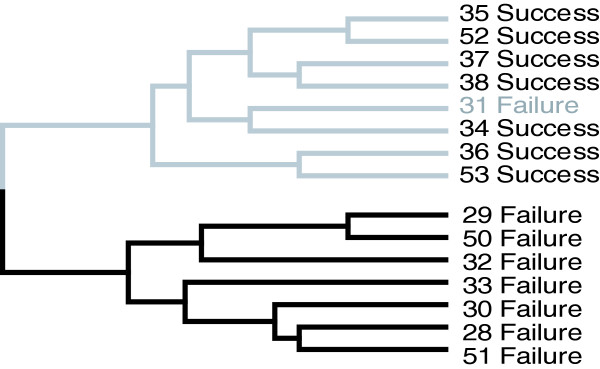
Detection of a sub-grouping of AML samples that clearly separates "Failure" *vs*. "Success" cases along with supporting genes. Note that informative genes which support this separation are selected solely based on their discrimination capacity with respect to AML *vs*. ALL classification and not for the discrimination of "Failure" *vs*. "Success" samples.

In addition to prediction accuracy, higher order genes may reveal a general tendency of samples to cluster in sub-groups within a given category. A characteristic example is given by the separation of T-cell from B-cell samples within the ALL leukemia class. Note that this separation is achieved using informative genes selected according to their discrimination capacity with respect to the original class distinctions, in this case AML *vs*. ALL. Interestingly, among the seven identified genes that support the T-cell *vs*. B-cell separation (see Table 3 in [Additional file 1]), gene X00437 corresponds to a protein that specifies part of the human T-cell receptor [23]. While it is expected that such a gene would support T-cell *vs*. B-cell discrimination, it is not intuitive that it would be selected as an AML *vs*. ALL classifier. In a similar context, our technique is able to separate "Failure" from "Success" AML samples with high accuracy as well as identify the genes that achieve this separation (see Table 2 in [Additional file 1]). Comparable discrimination results were previously achieved with other methods but only when treating these samples as distinct classes and selecting genes that specifically discriminate between the two [26,60] (also see Figure 3 and Figure 4 in [Additional file 1]).

In conclusion, this work describes a new method for the identification of informative genes that takes into account inherent genetic variation in disease samples which may be characteristic of certain sub-groups within a disease category. This relatively simple approach, in conjunction with a committee voting classifier allows for improved class prediction as well as identification of interesting disease sub-groups. More importantly, our method allows the detection of marker genes that support these sub-groupings, thus possibly shedding some light on the underlying molecular mechanisms involved in disease related processes and providing a new tool that may facilitate efforts towards individualized medicine.

## Methods

### Identification of informative genes and construction of gene pool

The proposed algorithm uses a training set comprised of labeled samples belonging to two categories (0 or 1) to construct a pool of informative genes that exhibit Consistent Expression Regions (CERs). CERs are defined as the intervals enclosing the expression (sorted in ascending order) of a given gene in a significant number of training samples which belong to the same category. Examples of informative genes and associated CERs are shown in Figure 2. The consistency of a CER is given by the fraction of these majority samples in CER, normalized by the size of their respective category. Only genes with at least one CER whose consistency value is greater than a statistically defined threshold, *p*_*s*_, are used to construct the pool. The order of each informative gene with respect to a category (0 or 1) reflects the number of class-specific CERs it consists of (for more details about the estimation of consistency thresholds and gene orders see [Additional file 1]).

The outcome of this step is the identification of category-specific classifiers formed by expression regions in the profiles of selected genes as opposed to a single expression threshold defined by most existing feature selection methods. As a result, a gene exhibiting a class-specific CER can be used to reliably assign a label of the same class to any new sample in which its expression lies within the boundaries of this CER. Note that any informative gene can produce several different regional classifiers, according to its CER assortment.

The contribution of these thresholded expression regions could be twofold. First, their mapping to a limited sample number of the same class may provide insights about the complexity of a given disease category. For example, CERs of the same category may reflect differences in the order and/or extent that various cancer-associated molecular processes are utilized to induce qualitatively the same phenotype but with a different gene expression pattern. It is thus conceivable that CERs can detect subgroups within a single class. Second, the classification accuracy of these regions, which is overlooked by existing methods focusing on the expression profile of a gene as a whole, can be used to construct a potentially more powerful classifier that takes into account the individuality of different samples.

### Class prediction using CERs and hierarchical clustering

To classify unseen samples into their respective categories, the method combines subsets of informative genes to form an aggregate classifier. For a two-class problem, the aggregate classifier consists of two -possibly overlapping- lists of informative genes. Each list consists of the set of informative genes that contain CERs specific to each class. If an informative gene contains at least two CERs, each corresponding to a different class (Figure 2), it serves as a classifier for both classes. For the categorization of each new sample the method proceeds as follows: first, the subsets of genes that are able to predict its category are retrieved from each class-specific list. Their respective CERs are then used to assign a class label to the new sample thus generating two lists of 0 and 1 votes, respectively. At this stage, the votes correspond to the informative genes containing the CER and not the CER itself. The procedure is repeated for all unseen samples and the class assignments for each sample are fed to a modified Manhattan distance to estimate dissimilarity scores between samples. Specifically, the distance between two samples *a *and *b *is defined as:

*D*(*a*, *b*) = *T *- *C*(*a*, *b*)     (1)

where *T *is the total number of informative genes which constitute the aggregate classifier and *C*(*a*, *b*) is the number of genes that give the same vote (0 or 1) for both samples *a *and *b*. Alternatively, a similarity score between samples a and b is given by *C*(*a*, *b*). The determination of similarities between samples is graphically illustrated in Figure 3. Finally, the dissimilarity scores between all samples are fed in the publicly available phylogenetic software MEGA2 [61] to build a hierarchical tree. The method's performance is measured as its discrimination capacity on the set of unseen samples.

#### Comparison to Entropy-based methods

Since the proposed method may sound similar to Entropy-based discretization methods frequently used in machine learning problems, we include a comparison between these two approaches.

In an information-based framework, Shannon's entropy [62] can be used for the evaluation of the information content of a given gene. The entropy of a particular k class input (k possible states) is given by the formula:

H=−∑i=1kpilog⁡2(pi)     (2)
 MathType@MTEF@5@5@+=feaafiart1ev1aaatCvAUfKttLearuWrP9MDH5MBPbIqV92AaeXatLxBI9gBaebbnrfifHhDYfgasaacH8akY=wiFfYdH8Gipec8Eeeu0xXdbba9frFj0=OqFfea0dXdd9vqai=hGuQ8kuc9pgc9s8qqaq=dirpe0xb9q8qiLsFr0=vr0=vr0dc8meaabaqaciaacaGaaeqabaqabeGadaaakeaacqWGibascqGH9aqpcqGHsisldaaeWbqaaiabdchaWnaaBaaaleaacqWGPbqAaeqaaOGagiiBaWMaei4Ba8Maei4zaC2aaSbaaSqaaiabikdaYaqabaaabaGaemyAaKMaeyypa0JaeGymaedabaGaem4AaSganiabggHiLdGccqGGOaakcqWGWbaCdaWgaaWcbaGaemyAaKgabeaakiabcMcaPiaaxMaacaWLjaWaaeWaceaacqaIYaGmaiaawIcacaGLPaaaaaa@474C@

where *p*_*i *_corresponds to the probability of the state *i*. In the analysis presented here *k *equals 2, since all datasets were broken down to two-class discrimination problems.

There are several entropy-based discretization methods, including the Maximum entropy [63], D2 [64], and Entropy-MDLC [65] among others and quite a few have been used in microarray data analysis [24,66,67]. Maximum entropy discretizes the continuous variables (in this case the gene expression profiles) according to a minimum loss of information criterion. D2 partitions the set of values into two subsets that maximize the information gain after the binary partition. Entropy-MDLC uses the class information entropy of candidate partitions to select threshold boundaries for discretization. It finds a single threshold that minimizes the entropy function over all possible thresholds and recursively applies this strategy to both induced partitions. The recursive discretization is terminated when the Minimum Description Length criterion is satisfied. The Entropy-MDLC method, which allows the generation of multiple intervals, is more similar to the method introduced in this paper. However, several differences can be pointed out between entropy-based approaches and ours.

1. Entropy-based methods consider all possible states (classes) of the input data in order to estimate discretization thresholds and identify informative genes. On the contrary, the method presented here detects genes whose expression within a well defined region consistently maps to a single class, without taking into account the remaining classes.

2. Entropy-based methods usually search for a single expression threshold that minimizes the entropy of a discretized gene. Although multi-interval discretization can be achieved with iterative application of entropy-based minimization methods [65], such an approach is of high computational cost. The basic idea of this method is to partition a range of real values into a number of disjoint intervals such that the entropy of the intervals is minimal. However, this method also considers minimization criteria that involve both classes for each interval.

3. Finally, the method presented here does not utilize any minimization criterion but searches for statistically significant homogeneous regions (CERs) in which no other state can occur as opposed to entropy-based methods where a few instances of other states are allowed.

### Detection of sample sub-groups

Identification of sub-groups within a given disease class can be of major importance as it may pinpoint patient subcategories that respond differentially to a given treatment. To detect such sub-groups, the method utilizes informative genes that inherently separate a main class into two or more clusters by grouping different subsets of samples in different CERs. An example is given by gene *VII *in Figure 2, whose expression profile separates *class 1 *samples into two distinct sub-groups. Down-regulation of this gene is characteristic of the first while up-regulation is characteristic of the second sub-group. For the detection of within class sub-groups, the method combines all higher order informative genes (i.e. genes that contain more than one CER corresponding to the same class) or just a selected subset of them.

#### Using all genes with multiple single-class CERs

In this approach, all informative genes that contain at least two CERs specific to the same class (*n *> 1) are utilized. For each informative gene, samples that lie within different CERs are assigned to different sub-groups, using a voting scheme similar to that of the class prediction task. However, in contrast to the class prediction task, CERs of the same gene now offer a different vote which can take a value raging from 0 to the gene order. Resulting voting lists are then used along with the modified Manhattan distance to construct a dendrogram. This approach is particularly suitable for datasets in which the actual number of class categories is larger than originally suggested, as for example the classification of ALL/MLL/AML leukemia samples shown in Figure 6.

#### Using a tight set of genes

**Figure 5:**
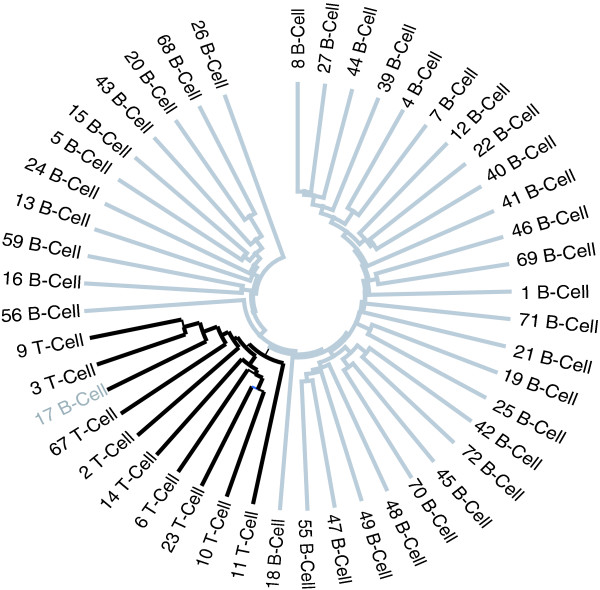
Discrimination of T-cell *vs*. B-cell lymphomas within the ALL leukemia samples using higher order genes. T-cell samples are clustered within a single branch, clearly separated from B-cell samples.

**Figure 6:**
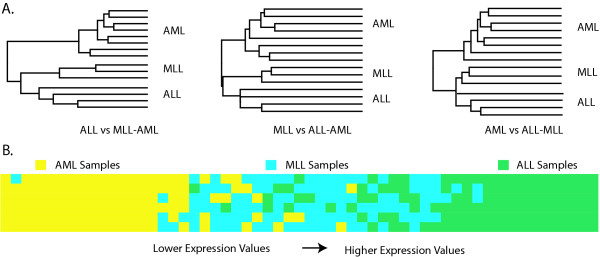
Separation of AML, MLL and ALL leukemia samples into three distinct clusters and supporting genes. A. Note that all three dendrograms assign MLL samples closer to ALL samples. B. Expression profiles of six of the most important genes that support this clustering. As evident from the figure, different expression intervals correspond to the three different classes for all six genes. More information about these genes can be found in Table 5 of [Additional file 1].

**Figure 7:**
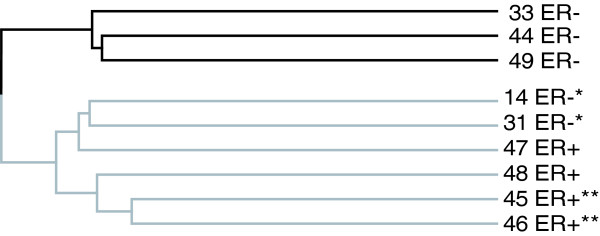
Separation of *ER*^- ^and *ER*^+ ^breast cancer samples. According to the immunoblotting classification, our method has two misclassified samples, namely 14 and 31. However, both samples are placed at the furthest end of the *ER*^+ ^cluster and are joined in a single branch, possibly indicating an intermediate disease stage.

In the second approach, only genes of the same order are used to identify sample sub-groups (as shown in Figures 4 and 5). Reversely, the method identifies genes that support a pre-existing sub-clustering of samples. These sub-groups, which may be irrelevant to the original classification, are often supported by only a small -*tight*- set of genes. A *tight *set consists of same-order genes whose expression in the same -significantly large- set of samples is bounded by exactly one CER per gene. More importantly, their expression in the remaining samples must range over several other CERs. The reasoning for this second constraint is that a gene which clusters in one region samples that lie in various regions of other genes has a tendency to distract the grouping achieved by these genes and should thus be omitted from the set. Notice that this procedure is applied to each class separately and thus genes used are all specific to the same class. For a more detailed explanation regarding the construction of *tight *sets of genes, see [Additional file 1].

#### Comparison to bi-clustering methods

The approach described above for the identification of sample and gene subgroups is fundamentally different from existing bi-clustering methods. Bi-clustering methods allow for the identification of sets of genes that share compatible expression patterns across subsets of samples. These methods group samples and genes simultaneously. According to [68], if *A *is an expression matrix, with *X *genes and *Y *conditions then *a*_*ij *_represents the expression of gene *i *at condition *j*. *I *⊂ *X *and *J *⊂ *Y *denote a subset of genes and conditions respectively. The pair (*I*, *J*) specifies a submatrix *A*_*IJ *_or a bi-cluster *A*_*IJ *_and *H*(*I*, *J*) represents the following mean squared residue score:

H(I,J)=1|I||J|∑i∈I,j∈J(aij−aiJ−aIj+aIJ)2     (3)
 MathType@MTEF@5@5@+=feaafiart1ev1aaatCvAUfKttLearuWrP9MDH5MBPbIqV92AaeXatLxBI9gBaebbnrfifHhDYfgasaacH8akY=wiFfYdH8Gipec8Eeeu0xXdbba9frFj0=OqFfea0dXdd9vqai=hGuQ8kuc9pgc9s8qqaq=dirpe0xb9q8qiLsFr0=vr0=vr0dc8meaabaqaciaacaGaaeqabaqabeGadaaakeaacqWGibascqGGOaakcqWGjbqscqGGSaalcqWGkbGscqGGPaqkcqGH9aqpdaWcaaqaaiabigdaXaqaaiabcYha8jabdMeajjabcYha8jabcYha8jabdQeakjabcYha8baadaaeqbqaaiabcIcaOiabdggaHnaaBaaaleaacqWGPbqAcqWGQbGAaeqaaOGaeyOeI0Iaemyyae2aaSbaaSqaaiabdMgaPjabdQeakbqabaGccqGHsislcqWGHbqydaWgaaWcbaGaemysaKKaemOAaOgabeaakiabgUcaRiabdggaHnaaBaaaleaacqWGjbqscqWGkbGsaeqaaOGaeiykaKYaaWbaaSqabeaacqaIYaGmaaaabaGaemyAaKMaeyicI4SaemysaKKaeiilaWIaemOAaOMaeyicI4SaemOsaOeabeqdcqGHris5aOGaaCzcaiaaxMaadaqadiqaaiabiodaZaGaayjkaiaawMcaaaaa@60FB@

where *a*_*iJ*_, *a*_*Ij *_denote the average of the *i*^*th *^gene and *j*^*th *^condition respectively and *a*_*IJ *_the average of all elements in the bi-cluster. This score is always positive, and the goal of the analysis is to find the largest submatrix with the lowest score or to find the bi-cluster whose score is lower than a certain threshold. This submatrix denotes a subset of genes with similar "behavior" in a subset of conditions. In a similar concept, the method presented here aims to connect a certain subset of genes with a specific subset of conditions in order to detect subgroups of conditions (or samples). However the two methods use totaly different criteria for the identification of similarly "behaving" genes.

1. In bi-clustering methods, selected genes within a bi-cluster must share similar expression profiles. In the proposed method these genes are only required to map approximately the same set of samples within a single CER, irrespectively of the expression values contained in this CER. We term these regions "significantly overlapping."

2. In addition to this similarity criterion, the proposed method demands that selected genes do not contain more than one significantly overlapping CER for a specific subset of samples. It is however possible to have several significantly overlapping CERs within the same subset of genes as long as they contain distinct subsets of samples. As a result, a set of genes containing two clusters of CERs can thus represent two different sample sub-groups.

3. The above criteria identify subsets of genes each of which group together a subset of samples in a single CER, irrespectively of co-expression constraints. If CERs were thought as independent features, the proposed method would resemble bi-clustering approaches except for the co-expression requirement which is not a prerequisite here.

## Authors' contributions

P. Pavlidis developed and tested the algorithm in publicly available microarray datasets under the guidance and supervision of P. Poirazi. P. Pavlidis and P. Poirazi designed and drafted the manuscript. All authors read and approved the final manuscript. The software used in this work can be found at [69].

## Supplementary Material

Additional File 1 **Supp.doc, AML-ALL-overlap.pdf, Breast-Cancer-overlap.pdf, Lung-Cancer-overlap.pdf, AML-MLL-ALL-overlap.pdf, CNS-overlap.pdf**. The supplementary material of this article. Click here for file

## Figures and Tables

**Figure 1:**
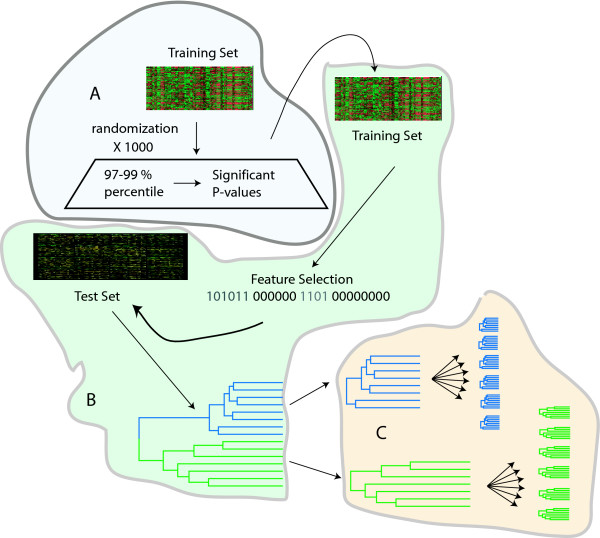
Flowchart of the proposed method. (A) A randomization approach is used to identify homogeneous expression regions of statistically significant length within each gene. (B) Genes that contain such regions are used to discriminate between class categories via hierarchical clustering. (C) Detection of sub-groups within each class is then based on informative gene clustering.

**Table 1:** Comparison of Proposed Method Performance against Existing Methods on Publicly Available Datasets. As shown in the table, the proposed method achieves a high classification performance on all datasets tested. In particular, the performance is superior to that of the referenced method in 3/6 datasets and matches that of the referenced method in the remaining 3 datasets. The last column shows the ratio of genes selected by both ours and the cross reference method over the total number of gene-features in the cross reference method. Abbreviations: *S2N*: Signal to Noise, *CC*: Correlation Coefficient, *NA*: Neighborhood Analysis, *FA*: Factor Analysis, *2-tail T*: 2-Tail Student test, *ER*: Expression Ratio, *K-NN*: K-Nearest Neighbors. *Outlier samples for this dataset were omitted from the classification in both the reference and our method.

**Cross Reference Method**				**Our Method**				
Data Set	Performance		Selection/Classification	Performance		Sensitivity	Specificity	**Common genes**
	CV	Test		CV	Test			

AML/ALL	-	29/34	S2N/NA	-	33/34	90.9%	100%	48/50
Breast Cancer	47/49	5/5*	CC/FA	48/49	5/5*	100%*	100%*	81/100
Lung Cancer	-	148/149	2-tail T/ER	-	148/149	93.3%	100%	8/8
AML/MLL/ALL	54/57	14/15	CC/K-NN	56/5	4/4, 3/3, 8/8	-	-	14/45
CNS	47/60	-	S2N/K-NN	55/60	-	85.7%	94.9%	22/100
Lymph Node	31/34	-	CC/FA	31/34	-	83.3%	95.4%	N/A
