# Supplementary material for: Individualized markers optimize class prediction of microarray data
Source: BMC Bioinformatics. 2006 Jul 14;7:345. doi: 10.1186/1471-2105-7-345 (PMC1569876; doi:10.1186/1471-2105-7-345)
Supplement: Additional File 1 — Supp.doc, AML-ALL-overlap.pdf, Breast-Cancer-overlap.pdf, Lung-Cancer-overlap.pdf, AML-MLL-ALL-overlap.pdf, CNS-overlap.pdf. The supplementary material of this article. [file 1471-2105-7-345-S1.doc]

Online Supplement for the manuscript

Individualized markers optimize class prediction of microarray data

**Pavlos Pavlidis^1,2^ and Panayiota Poirazi^1,*^**

[Methods 2](#__RefHeading___Toc102237494)

[Feature Selection 2](#__RefHeading___Toc102237495)

[Classification 3](#__RefHeading___Toc102237496)

[Distance Measurement 3](#__RefHeading___Toc102237497)

[Hierarchical Clustering 4](#__RefHeading___Toc102237498)

[Subgroups 4](#__RefHeading___Toc102237499)

[Identification of subgroups using a “tight” set of genes 4](#__RefHeading___Toc102237500)

[Results 6](#__RefHeading___Toc102237501)

[Classification Results **Error! Bookmark not defined.**](#__RefHeading___Toc102237502)

[Leave-one-Out Cross Validation 7](#__RefHeading___Toc102237503)

[LOOCV Results from Leukemia Dataset (Golub, Slonim et al. 1999) 7](#__RefHeading___Toc102237504)

[10](#__RefHeading___Toc102237505)

[Additional Datasets 6](#__RefHeading___Toc102237506)

[Lymph Node Results 6](#__RefHeading___Toc102237507)

[Lung Cancer Results 6](#__RefHeading___Toc102237508)

[Discussion **Error! Bookmark not defined.**](#__RefHeading___Toc102237509)

# Methods

### Feature Selection Procedure

Each gene expression data set is split into Training and Test sets according to respective reference publications. A Training set T, consists of M samples and N genes. For simplicity reasons we describe the feature selection method on a two class problem. In this case we arbitrarily assign labels 0 and 1 to samples that belong in the first and second class respectively. For each gene ***g***, **E_g_ = (e_1_, e_2_, …, e_m_)** contains its expression values across all samples and **V_g_ = (v_1_, v_2_, …, v_m_)** contains the respective class labels for these samples. To test the discriminatory power of each gene, we use its sorted expression profile **E_g_**, and the rearranged labeling vector **V_g_**, which reflects the new ordering of expression values, as shown in Table 1. This sorting approach was inspired by the work of Ben-Dor et al. (Ben-Dor, Friedman et al. 2001)

Table 1

| Samples | expression value | class Label |
| --- | --- | --- |
| e_1_ | 10 | 0 |
| e_2_ | 3 | 0 |
| e_3_ | 52 | 1 |
| e_4_ | 27 | 1 |
| e_5_ | 45 | 0 |

sorting

**E_g_** = (10,3,52 27,45) **E_g_** = (3,10,27,45,52)

**V_g_** = (0, 0, 1, 1, 0) **V_g_** = (0, 0, 1, 0, 1)

For the detection of the most informative genes in the Training Set we scan each labeling vector and search for one or multiple homogeneous regions (Figure 2A in paper). A value$P_{i}^{j}$, (see Methods section in paper for the definition of$P_{i}^{j}$) is used to determine a threshold for the consistency of the regions. If the consistency of the respective region is greater than the $P_{i}^{j}$value, the vector **V**g contains an informative region R.

## Estimation of Consistency Thresholds

In order to identify CERs with statistically significant classification accuracy, we use a consistency threshold. A *consistency threshold* value ($P_{i}^{j}$) for an informative gene **g** which contains ***j*** expression regions specific for class ***i****,* is defined as: the minimum consistency percentage for which the probability of finding a region **R** in a ***j^th^*** *order* gene, for the ***i^th^*** class category, with consistency equal or greater than this threshold in a randomly labeled dataset is less than p_s_, where p_s_ ranges from 1%-3%. *Consistency thresholds*$P_{i}^{j}$, *i=0,1* and *j=1,2,…n* are calculated using a statistical approach in which the labels of all samples in the training set are randomly permuted 1000 times and potentially consistent expression regions for all genes are identified. For a given class *i* and for every *order* *j*, the smallest consistency percentages with probability less than p_s_ in the right side of the tail of the resulting distribution over all genes are selected. The maximum values of these selected percentages represent the *consistency thresholds*$P_{i}^{j}$, for each *i* and for each *j*. For example, for a p_s_ value ranging from 1%-3%, in a dataset containing 1000 consistent regions of the first order, 10 – 30 of these regions will have been formed at random . Although this number might seem large, it does not affect our results since not all CERs in the pool are used to classify a given sample. On the contrary, this approach ensures that a maximum number of potentially useful genes are selected.

### Classification Procedure

#### Distance Metric

Any clustering or grouping technique involves the determination of distance measurement i.e. the identification of close (or related) samples. Distance or similarity functions are mathematical expressions that determine what is considered “close” (Dudoit S. and Gentleman R., 2002). The selection of the most appropriate distance or similarity function is a difficult task as it greatly affects the resulting dendrogram topology. In agglomerative hierarchical approaches (bottom – up approaches) the most widely used distance metrics include: 1. Euclidean distance, 2. Mahalanobis distance, 3. Manhattan distance, 4. Canberra distance.

The distance metric we use is given by the equation:

$D(a,b)=T-C(a,b)$ (1)

where a and b are two sample vectors, $T$ is the total number of informative genes which constitute the classifier and$C(a,b)$ is the number of genes that give the same vote for both samples *a* and *b***.** This distance measurement is similar to the Manhattan distance between two vectors x, y:

$d_{Mn}(x,y)=\sum_{i} \left|x_{i}-y_{i}\right|$ (2)

In our case $x_{i}$represents the vote of i^th^ classifier (it can be 1 or 0). We use a metric similar to Manhattan distance because this function is robust to outliers (Heydebreck 2003).

#### Hierarchical Clustering

We use the UPGMA algorithm to build a dendrogram of our classification results as it is a more intuitive and practical tool for visualization. The UPGMA method does not suffer from randomness in the initial conditions as it forms the first node between the sample pair with the minimum distance value. This simple method was first applied to gene expression data by (Eisen, Spellman et al. 1998). We used an algorithm written by Sestsof P. (Sestoft 1999) which implements the UPGMA and Neighbor-Joining Algorithm and the publicly available phylogenetic software MEGA2 (Kumar, Tamura et al. 2001). In cases where we wanted to know the branch lengths, we use the Neighbor-Joining method (Saitou and Nei 1987).

### Subgroups

#### Identification of subgroups using a “tight” set of genes

The following two definitions are needed for this procedure:

1. $V'_{i}(g)$ is the respective sorted vector containing sample-indexes (instead of labels) for gene ***g***.
2. S_ij_ is defined as the similarity between two regions *i* and *j* of two different genes. It is defined as the percentage of common sample indexes out of all distinct indexes contained in the two regions.

The following figure shows the similarity values for pair-wise comparisons between three different genes of the same order (2^nd^ order genes).


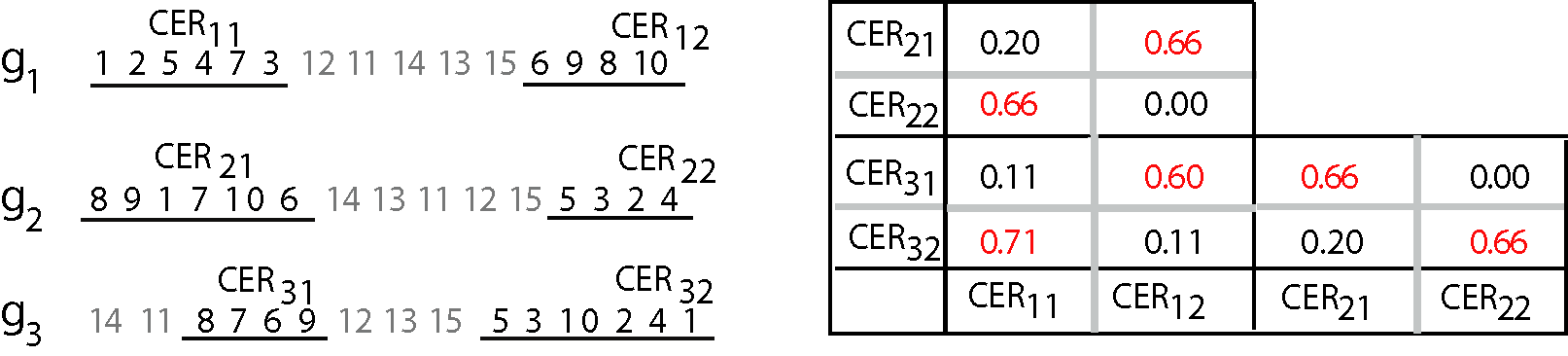


Figure 1: Similarity estimation for three second order genes g_1_, g_2_, g_3_. The numbers in each vector correspond to sample indexes. CER_k,l_ represents the Consistent Expression Region *k* of gene *l*. The matrix illustrates the similarity between two CERs of different genes. The values in red represent the maximum similarity for each comparison.

The first constraint for the identification of a “tight” set of marker-genes is that each gene’s CER must be - at least - S_eq_ % identical with *exactly one CER* from every other gene. The first prerequisite is not so crucial and typically for the datasets we used S_eq_ was 40% - 60%. The second constraint states that *for any other pair of CERs* *i* and *j* between two genes, except those that exceed the threshold S_eq_, the similarity S*_ij_* must be lower than S_un_. S_un_ is the maximum allowed similarity between each pair of CERs for which Similarity S_ij_ < S_eq_ and typically its value is less than 20%. The matrix in Figure 1 illustrates these constraints. The comparison between g_1_ and g_2_ results in a similarity sub-matrix inside the matrix (1^st^ and 2^nd^ row). The above prerequisites can be interpreted in the following way: in every row of this sub-matrix there must be exactly one cell with a value greater than S_eq_. These are the 2^nd^ cell, in the first row, and the 1^st^ cell in the second row. Furthermore, these two cells must belong to different columns of the sub-matrix. Otherwise one region of a gene is similar with more than one regions of the other gene. In addition, all the other cells inside this sub-matrix must have lower values than S_un_. So, for genes g_1_, g_2_, g_3_: if S_eq_ = 0.59 and S_un_ = 0.19, then these genes form a “tight” set.

The meaning of a “tight” set is the following: Among the informative genes having a predefined number of regions, we seek the genes that cluster, in one region, almost the same samples. More importantly, for every other pair of regions except the most similar ones, the sample overlapping must be very low. A gene that clusters in one region samples which are in different regions among other genes has a tendency to destruct the clustering that is achieved by the remaining genes and must be excluded.

#### **Leave-one-Out Cross Validation**

Leave-one-Out Cross Validation (LOOCV) is a model evaluation method based on “re-sampling”. Considering a dataset with N samples, the model is trained using N-1 samples and classifies the sample that is omitted from the training procedure. The procedure is repeated N times and the model’s performance is calculated.

## Results

### Additional Datasets

#### Lymph Node Results

The Lymph Node dataset (West, Blanchette et al. 2001) consists of 34 samples split in two classes. “Reported negative” tumors (22 LN-) are samples with no positive lymph nodes, whereas “Reported Positive” tumors (12 LN+) have at least three identifiably positive nodes. The significance percentage p_s_ = 2% results in the selection of 410, 195, 237, 120 first, second, third, and fourth order marker genes, respectively. Our method has a classification performance of 31/34 samples correctly predicted on this dataset.

#### Lung Cancer Results

Lung Cancer Dataset (Gordon, Jensen et al. 2002) is composed of 181 tissue samples. 31 were retrieved from malignant pleural mesothelioma of the lung (MPM) and 150 from adenocarcinoma (ADCA). The total number of genes is 12533. The training set contains 16 MPM and 16 ADCA. The rest 149 samples are used for testing. For a significance percentage p_s_ = 1%, 640 first order, 173 second order and 4 third order genes are selected. Our method achieves a classification performance of 148/149 samples correctly predicted.

#### Additional Results for AML-ALL dataset of (Golub, Slonim et al. 1999)

AML vs. ALL sample classification using the (Golub, Slonim et al. 1999) dataset. Figure 2 shows a hierarchical tree of our classification results. Our method makes one mistake, namely sample 63 which is classified as ALL but belongs to the AML class.


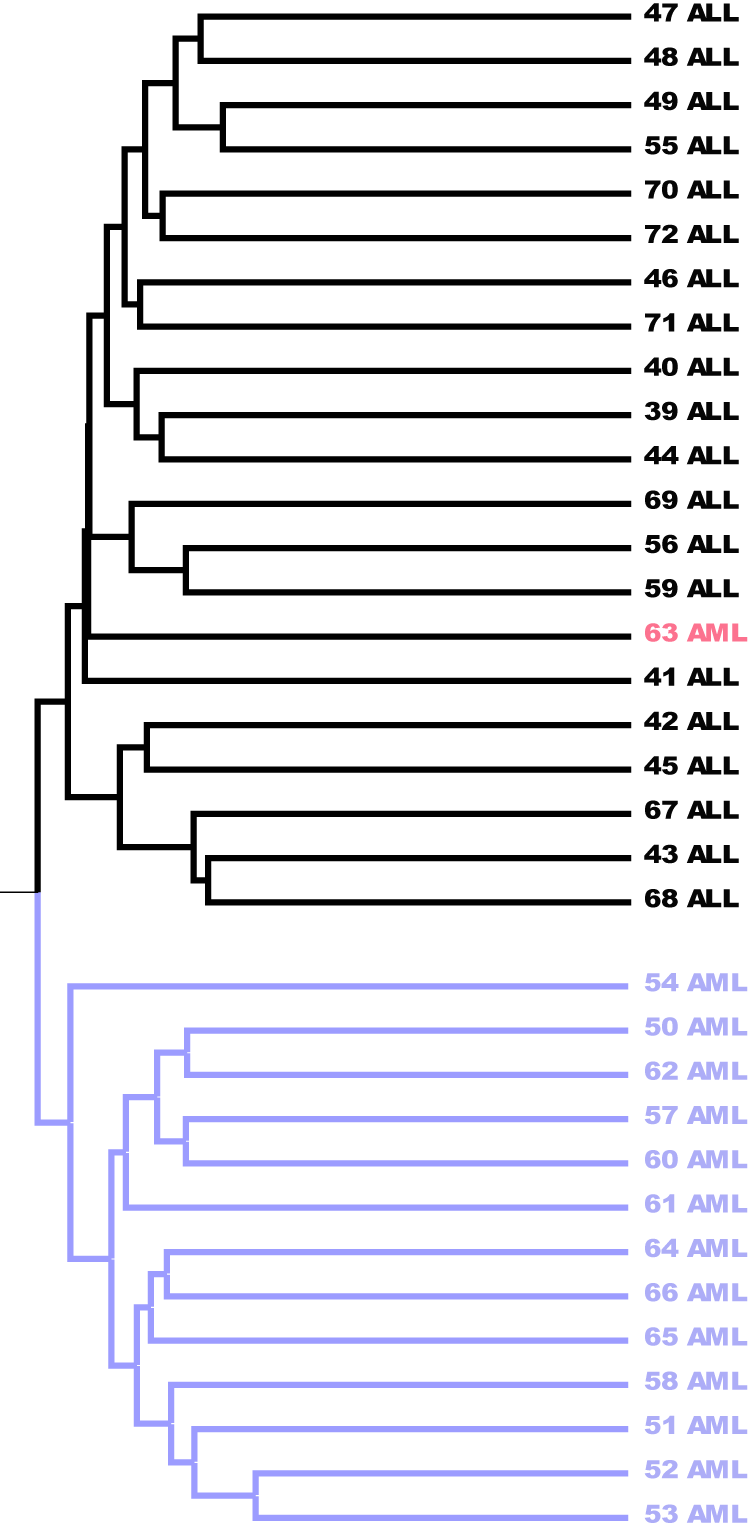


#### Figure 2: Hierarchical clustering depicting classification results for the AML-ALL Leukemia dataset of (Golub, Slonim et al. 1999). Our method has in one misclassified sample (63).

*LOOCV Results for Failure vs. Success Discrimination*

The following figures illustrate the generated dendrograms used for class prediction using LOOCV in the Leukemia Dataset with respect to Failure vs. Success discrimination.


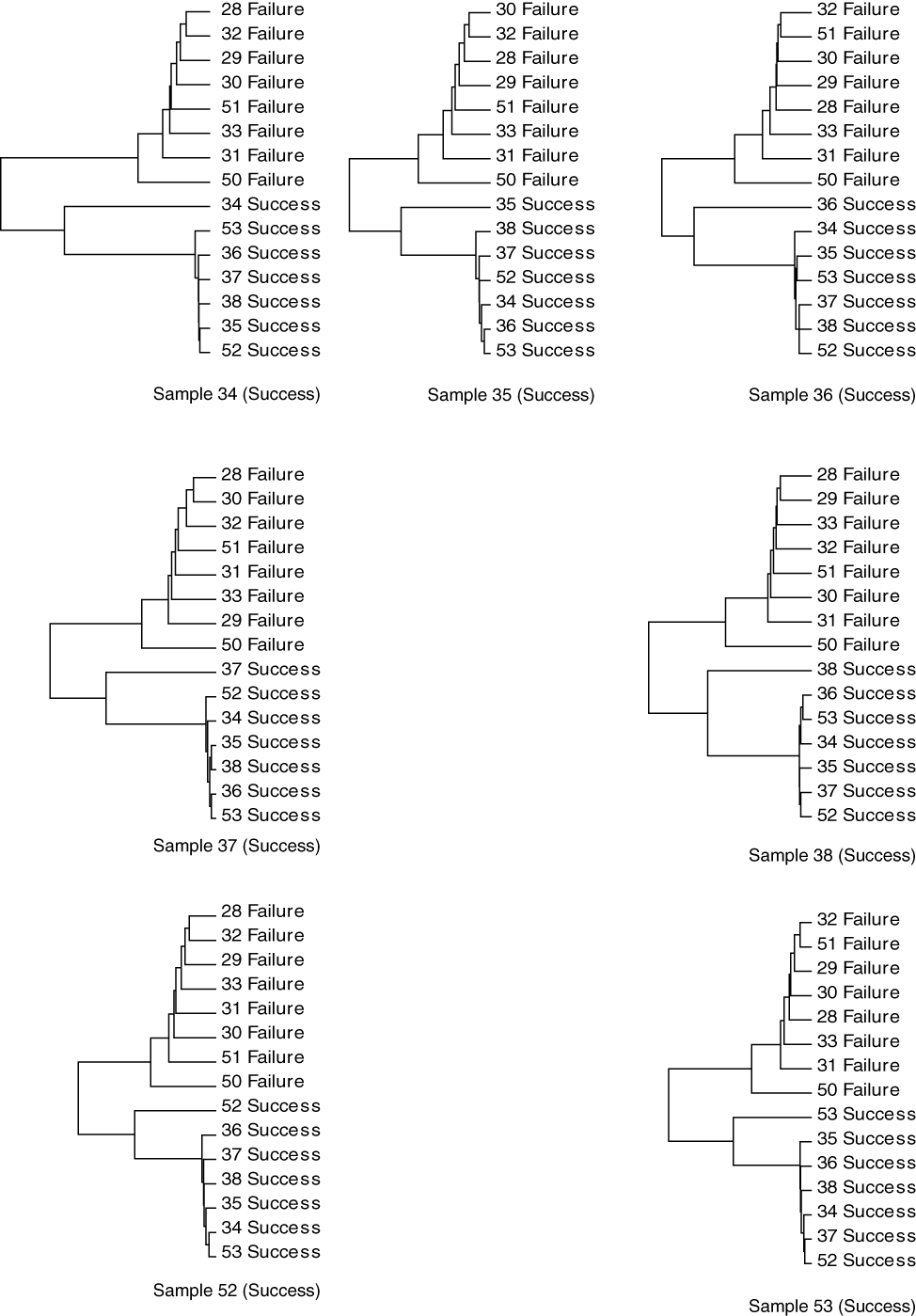


Figure 3: The dendrograms generated during LOOCV procedure for the “success” samples in Leukemia Dataset.

###
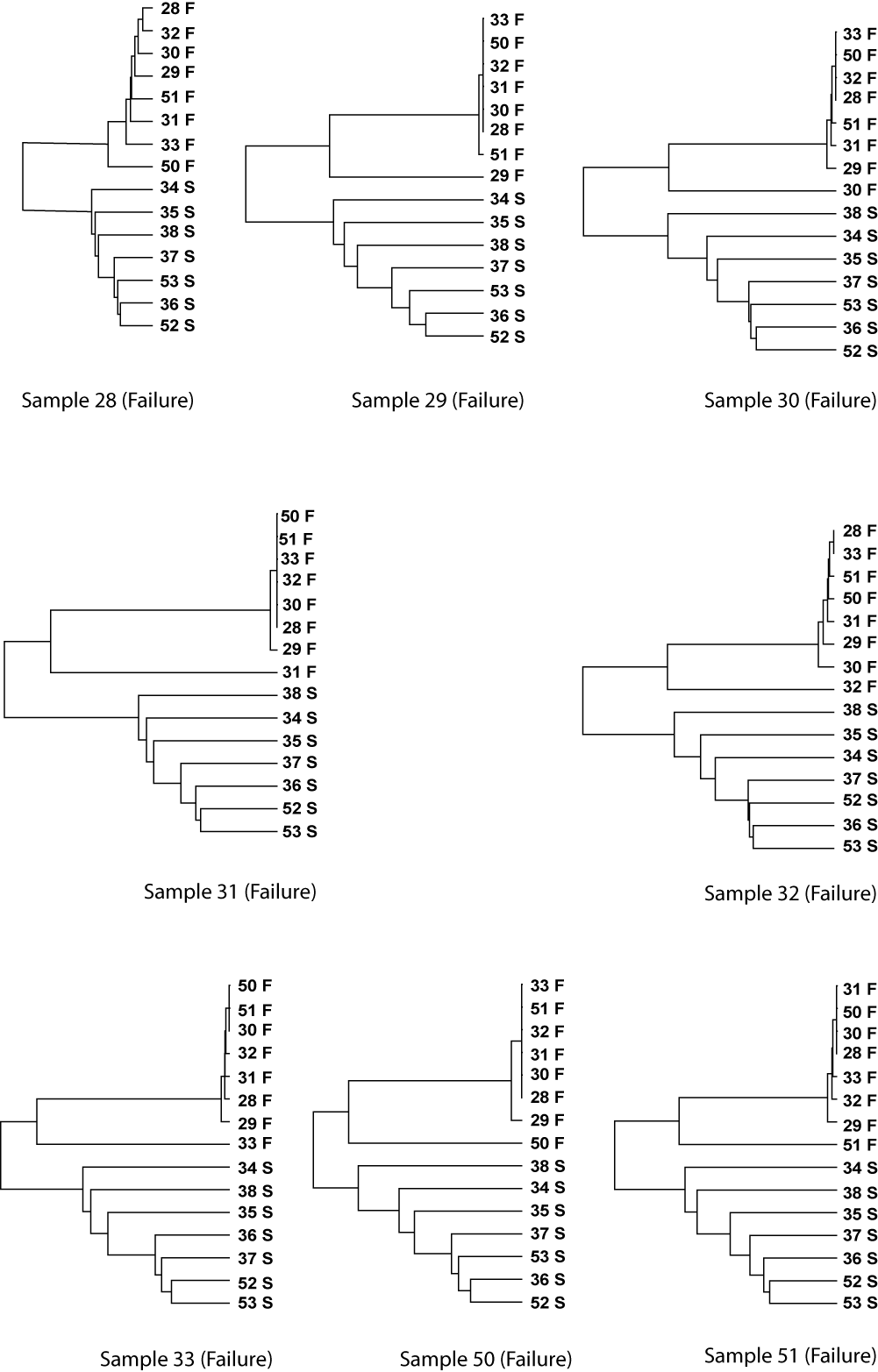


Figure 4: The dendrograms generated during LOOCV for the “failure” samples in Leukemia Dataset.


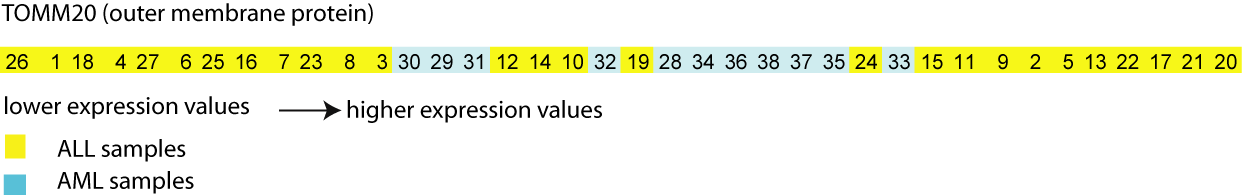


Figure 5: The expression profile for gene KIAA0016 reveals that both its lower *and* higher expression values are characteristic of ALL samples while most of the intermediate values are characteristic of AML samples

**Table 2: Genes that support the Failure vs. Success Discrimination of AML Samples**

| **Locus** | **Gene Name** | **Expression Level In AML / Higher Level in:** | [ExPASy](http://au.expasy.org/) Information |
| --- | --- | --- | --- |
| [U62136](http://au.expasy.org/cgi-bin/niceprot.pl?Q15819) | Putative enterocyte differentiation promoting factor mRNA, partial cds | Low / success | **FUNCTION**: Has no ubiquitin ligase activity on its own. The UBE2V2/UBE2N heterodimer catalyzes the synthesis of non-canonical poly-ubiquitin chains that are linked through Lys-63. This type of poly-ubiquitination does not lead to protein degradation by the proteasome. Mediates transcriptional activation of target genes. Plays a role in the control of progress through the cell cycle and differentiation. Plays a role in the error-free DNA repair pathway and contributes to the survival of cells after DNA damage.  **SUBUNIT**: Heterodimer with UBE2N. Binds CHFR.  **TISSUE SPECIFICITY**: Detected in placenta, colon, liver and skin. Detected at very low levels in most tissues.  **INDUCTION**: Up-regulated in cultured fresh blood cells upon treatment with vitamin D3.  **SIMILARITY**: Belongs to the ubiquitin-conjugating enzyme family. |
| [X74614](http://au.expasy.org/cgi-bin/niceprot.pl?Q14990) | ODF2 (allele 2) gene for outer dense fiber protein | High / success | **FUNCTION**: Component of the outer dense fibers (ODF) of spermatozoa. ODF are filamentous structures located on the outside of the axoneme in the midpiece and principal piece of the mammalian sperm tail and may help to maintain the passive elastic structures and elastic recoil of the sperm tail.  **SUBUNIT**: Interacts with SPAG4 (By similarity).  **TISSUE SPECIFICITY**: Testis.  **DOMAIN**: The C-terminal contains many C-X-P repeats. |
| [U80987](http://au.expasy.org/cgi-bin/niceprot.pl?Q99593) | Transcription factor TBX5 mRNA | Intermediate / success | **FUNCTION**: Involved in the transcriptional regulation of genes required for mesoderm differentiation. Probably plays a role in limb pattern formation.  **SUBCELLULAR LOCATION**: Nuclear (Potential).  **DISEASE**: Defects in TBX5 are the cause of Holt-Oram syndrome (HOS) [MIM:142900]. HOS is a developmental disorder affecting the heart and upper limbs. It is characterized by thumb anomaly and atrial septal defects.  **SIMILARITY**: Contains 1 T-box domain. |
| [S72043](http://au.expasy.org/cgi-bin/niceprot.pl?P25713) | growth inhibitory factor [human, brain, Genomic, 2015 nt] | High / success | **FUNCTION**: Binds heavy metals. Contains three zinc and three copper atoms per polypeptide chain and only a negligible amount of cadmium. Inhibits survival and neurite formation of cortical neurons in vitro.  **TISSUE SPECIFICITY:** Abundant in a subset of astrocytes in the normal human brain, but greatly reduced in the Alzheimer's disease (AD) brain.  **SIMILARITY**: Belongs to the metallothionein superfamily. Type 1 family [view classification]. |
| [M95610](http://au.expasy.org/cgi-bin/niceprot.pl?Q14055) | COL9A2 Collagen, type IX, alpha 2 | High / success | **FUNCTION**: Structural component of hyaline cartilage and vitreous of the eye.  **SUBUNIT**: Heterotrimer of an alpha 1(IX), an alpha 2(IX) and an alpha 3(IX) chain.  **PTM**: Covalently linked to the telopeptides of type II collagen by lysine-derived cross-links.  **PTM**: Prolines at the third position of the tripeptide repeating unit (G-X-Y) are hydroxylated in some or all of the chains.  **DISEASE**: Defects in COL9A2 are the cause of multiple epiphyseal dysplasia 2 (EDM2) [MIM:600204]. EDM is a generalized skeletal dysplasia associated with significant morbidity. Joint pain, joint deformity, waddling gait, and short stature are the main clinical signs and symptoms. EDM is broadly categorized into the more severe Fairbank and the milder Ribbing types. EDM2 inheritance is autosomal dominant.  **DISEASE**: Defects in COL9A2 may be a cause of susceptibility to intervertebral disc disease (IDD) [MIM:603932]. IDD is one of the most common musculo-skeletal disorders.  **SIMILARITY**: Belongs to the fibril-associated collagens with interrupted helices (FACIT) family. |
| [U60808](http://au.expasy.org/cgi-bin/niceprot.pl?Q92903) | CDP-diacylglycerol synthase (CDS) mRNA | Low / success | **FUNCTION**: Provides CDP-diacylglycerol an important precursor for the synthesis of phosphatidylinositol (PtdIns), phosphatidylglycerol, and cardiolipin. Overexpression may amplify cellular signaling responses from cytokines. May also play an important role in the signal transduction mechanism of retina and neural cells.  **CATALYTIC ACTIVITY**: CTP + phosphatidate = diphosphate + **CDP**-diacylglycerol.  **COFACTOR**: Magnesium (By similarity).  **PATHWAY**: Phospholipid biosynthesis.  **SUBCELLULAR LOCATION**: Integral membrane protein (Probable). Cytoplasmic aspect of the endoplasmic reticulum (By similarity).  **TISSUE SPECIFICITY**: Expressed in adult tissues such as placenta, brain, small intestine, ovary, testis and prostate. Highly expressed in fetal kidney, lung and brain. Lower level in fetal liver.  **SIMILARITY:** Belongs to the CDS family. |

**Table 3: Genes that support the T-Cell vs. B-Cell discrimination between ALL samples**

| **Locus** | **Gene Name** | **Higher expression in:** | [**ExPASy**](http://au.expasy.org/) **Information** |
| --- | --- | --- | --- |
| [D50918](http://au.expasy.org/cgi-bin/niceprot.pl?Q14141) (Swiss-Prot) | KIAA0128 gene, partial cds | T-Cell | **FUNCTION**: Involved in cytokinesis (Potential).  **SUBUNIT**: May assemble into a multicomponent structure. **SIMILARITY**: Belongs to the septin family. |
| [L05148](http://www.ncbi.nlm.nih.gov/entrez/query.fcgi?cmd=Retrieve&db=pubmed&dopt=Abstract&list_uids=8202713)  (PubMed) | Protein tyrosine kinase related mRNA sequence | T-Cell |  |
| [M13792](http://au.expasy.org/cgi-bin/niceprot.pl?P00813)  (Swiss-Prot) | ADA Adenosine deaminase | T-Cell | **CATALYTIC** **ACTIVITY**: Adenosine + H2O = inosine + NH3.  **TISSUE** **SPECIFICITY**: Found in all tissues, occurs in large amounts in T-lymphocytes and, at the time of weaning, in gastrointestinal tissues.  **POLYMORPHISM**: There is a common allele, ADA*2, also known as the ADA 2 allozyme.  **DISEASE**: Defects in ADA are a cause of autosomal recessive severe combined immuno-deficiency (SCID) [MIM:102700]..  **SIMILARITY**: Belongs to the adenosine and AMP deaminases family. |
| U02687 | Growth factor receptor tyrosine kinase (STK-1) mRNA | B-Cell |  |
| [L01087](http://au.expasy.org/cgi-bin/niceprot.pl?Q04759)  (Swiss-Prot) | PRKCQ Protein kinase C-theta  ` | T-Cell | **FUNCTION**: This is calcium-independent, phospholipid-dependent, serine- and threonine-specific enzyme.  **FUNCTION**: PKC is activated by diacylglycerol which in turn phosphorylates a range of cellular proteins. PKC also serves as the receptor for phorbol esters, a class of tumor promoters.  **SUBUNIT**: Interacts with TXNL2/PICOT.  **TISSUE** **SPECIFICITY**: Skeletal muscle, megakaryoblastic cells and platelets.  **SIMILARITY**: Belongs to the Ser/Thr protein kinase family. PKC subfamily.  **SIMILARITY**: Contains 2 phorbol-ester/DAG-type zinc fingers. |
| [M59807](http://au.expasy.org/cgi-bin/niceprot.pl?P24001)  (Swiss-Prot) | Natural Killer Cells Protein 4 Precursor | T-Cell | **FUNCTION**: May play a role in lymphocyte activation.  **SUBCELLULAR LOCATION**: Secreted.  **TISSUE** **SPECIFICITY**: Selectively expressed in lymphocytes.  **INDUCTION**: Expression increased after activation of T-cells by mitogens or activation of NK cells by IL-2. |
| [X00437](http://www.ncbi.nlm.nih.gov/entrez/query.fcgi?cmd=Retrieve&db=pubmed&dopt=Abstract&list_uids=6336315)  (PubMed) | TCRB T-cell receptor, beta cluster | T-Cell |  |

*Additional Results for Central Nervous System Dataset*


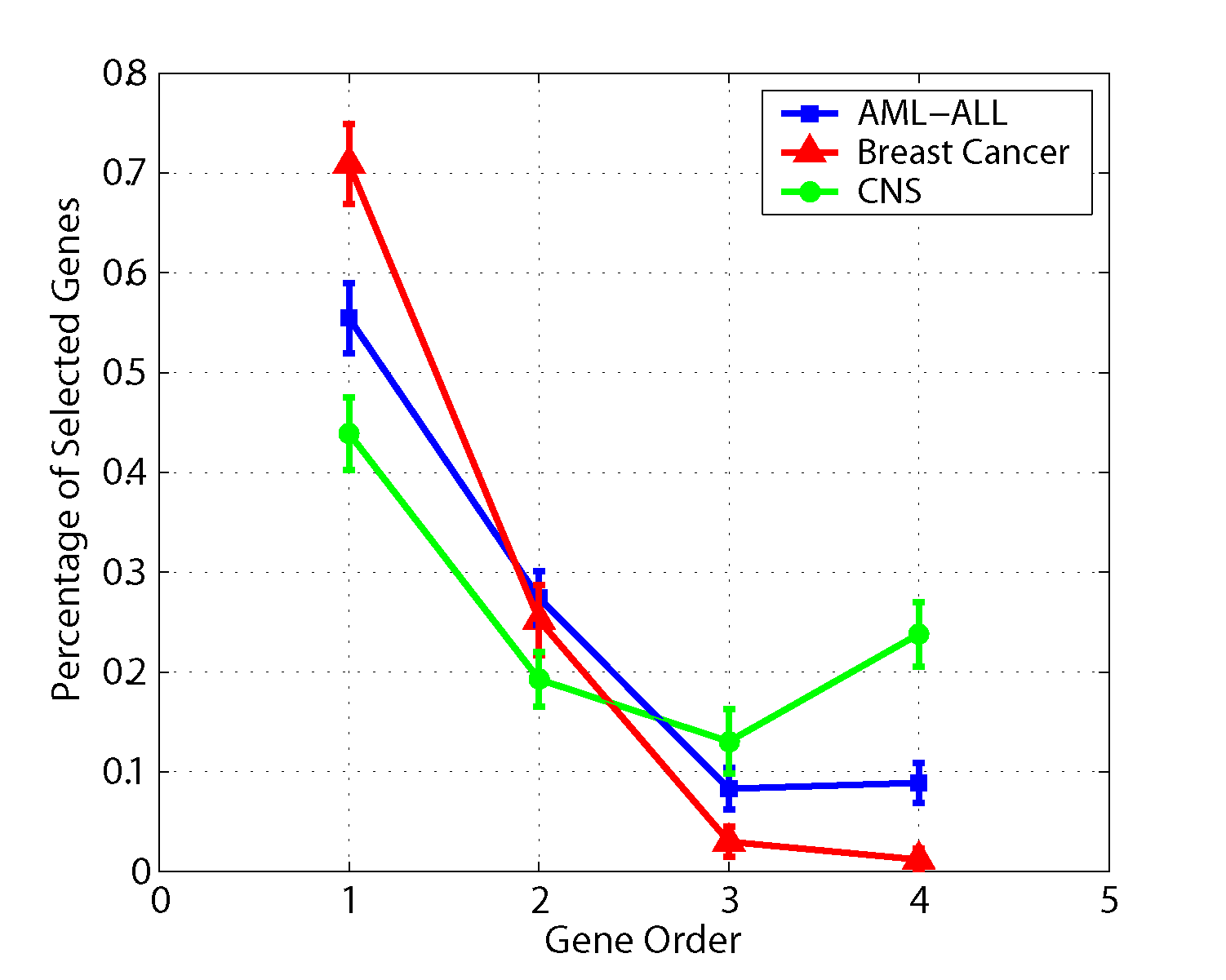


Figure 6: Distributions of 1^st^, 2^nd^, 3^rd^ and 4^th^ order informative genes in three data sets hybridized on the same chips. Average percentages and respective standard deviations shown in the figure are estimated using 20 random samplings of 50 features out of the total selected features for each dataset. Note the much higher content of 1^st^ order genes in the ALL-MLL and Breast Cancer datasets as compared to the Central Nervous System dataset. This is counteracted by the higher content of 4^th^ and 3^rd^ order genes in this dataset as opposed to the other two. Given that the standard deviation for the 3^rd^ and 4^th^ order genes is small, and 75% of the number of genes for each order is contained within the standard deviation limits, the presence of higher order genes in the CNS data is significant, suggesting an important discriminatory and perhaps biological role.

**Table 4: Examples of higher order (3^rd^ and 4^th^) genes in the CNS dataset**

| **Locus** | **Gene Name** | **Gene order:** | [**ExPASy**](http://au.expasy.org/) **Information** |
| --- | --- | --- | --- |
| [P36896](http://au.expasy.org/cgi-bin/get-sprot-entry?P36896) (Swiss-Prot) | **ACVR1B,** ACVRLK4 | 3^rd^ | **[FUNCTION](http://au.expasy.org/sprot/userman.html#CC_line)**: On ligand binding, forms a receptor complex consisting of two type II and two type I transmembrane serine/threonine kinases. Type II receptors phosphorylate and activate type I receptors which autophosphorylate, then bind and activate SMAD transcriptional regulators.  **[CATALYTIC ACTIVITY](http://au.expasy.org/sprot/userman.html#CC_line)**: ATP + [receptor-protein] = ADP + [receptor-protein] phosphate.  **[COFACTOR](http://au.expasy.org/sprot/userman.html#CC_line)**: Magnesium or manganese [(By similarity)](http://au.expasy.org/sprot/userman.html#Non-experimental_qualifier).  **[SUBUNIT](http://au.expasy.org/sprot/userman.html#CC_line)**: Interacts with AIP1. Part of a complex consisting of AIP1, ACVR2A, ACVR1B and SMAD3 [(By similarity)](http://au.expasy.org/sprot/userman.html#Non-experimental_qualifier).  **[SUBCELLULAR LOCATION](http://au.expasy.org/sprot/userman.html#CC_line)**: Membrane; single-pass type I membrane protein. |
| [P32970](http://au.expasy.org/cgi-bin/get-sprot-entry?P32970)  (Swiss-Prot) | **TNFSF7,** CD27L, CD27LG, CD70 | 3rd | **[FUNCTION](http://au.expasy.org/sprot/userman.html#CC_line)**: Cytokine that binds to TNFRSF7/CD27. Plays a role in T-cell activation. Induces the proliferation of costimulated T-cells and enhances the generation of cytolytic T-cells.  **[SUBUNIT](http://au.expasy.org/sprot/userman.html#CC_line)**: Homotrimer [(Probable)](http://au.expasy.org/sprot/userman.html#Non-experimental_qualifier).  **[SUBCELLULAR LOCATION](http://au.expasy.org/sprot/userman.html#CC_line)**: Membrane; single-pass type II membrane protein.  **[SIMILARITY](http://au.expasy.org/sprot/userman.html#CC_line)**: Belongs to the [tumor necrosis factor family](http://au.expasy.org/cgi-bin/get-similar?name=tumor necrosis factor family). |
| [P14778](http://au.expasy.org/cgi-bin/get-sprot-entry?P14778)  (Swiss-Prot) | **IL1R1,**  IL1R, IL1RA, IL1RT1 | 3rd | **[FUNCTION](http://au.expasy.org/sprot/userman.html#CC_line)**: Receptor for interleukin-1 alpha (IL-1A), beta (IL-1B), and interleukin-1 receptor antagonist protein (IL-1RA). Binding to the agonist leads to the activation of NF-kappa-B. Signaling involves formation of a ternary complex containing IL1RAP, TOLLIP, MYD88, and IRAK1 or IRAK2.  **[SUBUNIT](http://au.expasy.org/sprot/userman.html#CC_line)**: Binds IL1RAP.  **[INTERACTION](http://au.expasy.org/sprot/userman.html#CCBP)**: [Q86XR7](http://au.expasy.org/uniprot/Q86XR7):TRAM; NbExp=1; IntAct=[EBI-525905, EBI-525927](http://www.ebi.ac.uk/intact/search/do/search?binary=EBI-525905,EBI-525927);  **[SUBCELLULAR LOCATION](http://au.expasy.org/sprot/userman.html#CC_line)**: Membrane; single-pass type I membrane protein.  **[SIMILARITY](http://au.expasy.org/sprot/userman.html#CC_line)**: Belongs to the [interleukin-1 receptor family](http://au.expasy.org/cgi-bin/get-similar?name=interleukin-1 receptor family). Contains 3 [Ig-like C2-type (immunoglobulin-like) domains](http://au.expasy.org/cgi-bin/get-similar?name=Ig-like C2-type (immunoglobulin-like) domain). Contains 1 [TIR domain](http://au.expasy.org/cgi-bin/get-similar?name=TIR domain). |
| [Q00535](http://au.expasy.org/cgi-bin/get-sprot-entry?Q00535)  (Swiis-Prot) | **CDK5** | 3rd | **[FUNCTION](http://au.expasy.org/sprot/userman.html#CC_line)**: Probably involved in the control of the cell cycle. Interacts with d1 and d3-type G1 cyclins. Can phosphorylate histone H1, tau, MAP2 and NF-H and NF-M. Also interacts with p35 which activates the kinase.  **[CATALYTIC ACTIVITY](http://au.expasy.org/sprot/userman.html#CC_line)**: ATP + a protein = ADP + a phosphoprotein.  **[SUBUNIT](http://au.expasy.org/sprot/userman.html#CC_line)**: Heterodimer of a catalytic subunit and a regulatory subunit (p35). Found in a trimolecular complex with CABLES1 and ABL1. Interacts with CABLES1 [(By similarity)](http://au.expasy.org/sprot/userman.html#Non-experimental_qualifier).  **[SUBCELLULAR LOCATION](http://au.expasy.org/sprot/userman.html#CC_line)**: Cytoplasm. In axonal growth cone with extension to the peripheral lamellipodia [(By similarity)](http://au.expasy.org/sprot/userman.html#Non-experimental_qualifier).  **[SIMILARITY](http://au.expasy.org/sprot/userman.html#CC_line)**: Belongs to the [Ser/Thr protein kinase family. CDC2/CDKX subfamily](http://au.expasy.org/cgi-bin/get-similar?name=Ser%2FThr protein kinase family. CDC2%2FCDKX subfamily). Contains 1 [protein kinase domain](http://au.expasy.org/cgi-bin/get-similar?name=protein kinase domain). |
| [Q16836](http://au.expasy.org/cgi-bin/get-sprot-entry?Q16836)  (Swiss-Prot) | **HADHSC,** HAD, SCHAD | 4th | **[FUNCTION](http://au.expasy.org/sprot/userman.html#CC_line)**: Plays an essential role in the mitochondrial beta-oxidation of short chain fatty acids. Exerts it highest activity toward 3-hydroxybutyryl-CoA.  **[CATALYTIC ACTIVITY](http://au.expasy.org/sprot/userman.html#CC_line)**: (S)-3-hydroxyacyl-CoA + NAD^+^ = 3-oxoacyl-CoA + NADH.  **[PATHWAY](http://au.expasy.org/sprot/userman.html#CC_line)**: [Fatty acid beta-oxidation cycle; step 3](http://au.expasy.org/cgi-bin/get-similar?type=pathway&name=Fatty acid beta-oxidation cycle%3B step 3).  **[SUBUNIT](http://au.expasy.org/sprot/userman.html#CC_line)**: Homodimer.  **[SUBCELLULAR LOCATION](http://au.expasy.org/sprot/userman.html#CC_line)**: Mitochondrion; mitochondrial matrix. |
| [Q15392](http://au.expasy.org/cgi-bin/get-sprot-entry?Q15392)  (Swiss-Prot) | DHCR24, seladin-1 | 4th | **[FUNCTION](http://au.expasy.org/sprot/userman.html#CC_line)**: Catalyzes the reduction of the delta-24 double bond of sterol intermediates. Protects cells from oxidative stress by reducing caspase 3 activity during apoptosis induced by oxidative stress. Also protects against amyloid-beta peptide-induced apoptosis.  **[COFACTOR](http://au.expasy.org/sprot/userman.html#CC_line)**: FAD.  **[PATHWAY](http://au.expasy.org/sprot/userman.html#CC_line)**: [Cholesterol biosynthesis](http://au.expasy.org/cgi-bin/get-similar?type=pathway&name=Cholesterol biosynthesis).  **[SUBCELLULAR LOCATION](http://au.expasy.org/sprot/userman.html#CC_line)**: Endoplasmic reticulum; endoplasmic reticulum membrane; single-pass membrane protein. Golgi apparatus; Golgi membrane; single-pass membrane protein.  **[TISSUE SPECIFICITY](http://au.expasy.org/sprot/userman.html#CC_line)**: Highly expressed in brain and adrenal gland with moderate expression in liver, lung, spleen, prostate and spinal cord. Low expression in heart, uterus and prostate. Undetectable in blood cells. In the brain, strongly expressed in cortical regions, substantia nigra, caudate nucleus, hippocampus, medulla oblongata and pons. In brains affected by Alzheimer disease, expression in the inferior temporal lobe is substantially lower than in the frontal cortex.  **[DISEASE](http://au.expasy.org/sprot/userman.html#CC_line)**: Defects in DHCR24 are the cause of desmosterolosis [[MIM:602398](http://www.ncbi.nih.gov/entrez/dispomim.cgi?id=602398)]. It is a rare autosomal recessive disorder characterized by multiple congenital anomalies and elevated levels of the cholesterol precursor desmosterol in plasma, tissue, and cultured cells.  **[SIMILARITY](http://au.expasy.org/sprot/userman.html#CC_line)**: Belongs to the [FAD-binding oxidoreductase/transferase type 4 family](http://au.expasy.org/cgi-bin/get-similar?name=FAD-binding oxidoreductase%2Ftransferase type 4 family).  **[WEB RESOURCE](http://au.expasy.org/sprot/userman.html#CCDB)**: NAME=GeneReviews; URL="<http://www.genetests.org/query?gene=DHCR24>". |

*Additional Results for ALL-MLL-AML Leukemia Dataset*


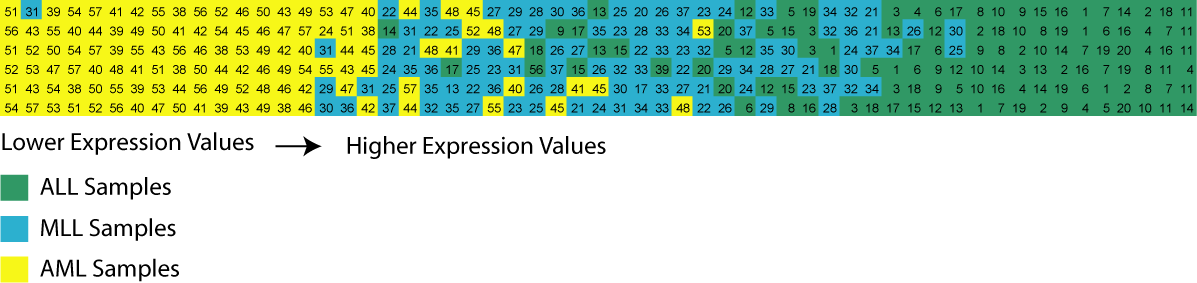


Figure 7: Expression profiles for 6 genes identified by our method that are differentially expressed between AML-MLL-ALL samples.

**Table 5: Genes that support the AML/MLL/ALL discrimination**

| **Locus** | **Gene Name** | **Expression order:** | [**ExPASy**](http://au.expasy.org/) **Information** |
| --- | --- | --- | --- |
| [P51532](http://au.expasy.org/cgi-bin/get-sprot-entry?P51532) (Swiss-Prot) | **SMARCA4,** BRG1, SNF2B, SNF2L4 | AML/MLL/ALL | **[FUNCTION](http://au.expasy.org/sprot/userman.html#CC_line)**: Transcriptional coactivator cooperating with nuclear hormone receptors to potentiate transcriptional activation.  **[SUBUNIT](http://au.expasy.org/sprot/userman.html#CC_line)**: Interacts with NR3C1, TOPBP1 and PGR. Component of the BAF53 complex, at least composed of BAF53A, RUVBL1, SMARCA4/BRG1, and TRRAP, which preferentially acetylates histone H4 (and H2A) within nucleosomes.  **[SUBCELLULAR LOCATION](http://au.expasy.org/sprot/userman.html#CC_line)**: Nucleus.  **[SIMILARITY](http://au.expasy.org/sprot/userman.html#CC_line)**: Belongs to the [SNF2/RAD54 helicase family](http://au.expasy.org/cgi-bin/get-similar?name=SNF2%2FRAD54 helicase family). Contains 1 [bromo domain](http://au.expasy.org/cgi-bin/get-similar?name=bromo domain). Contains 1 [helicase ATP-binding domain](http://au.expasy.org/cgi-bin/get-similar?name=helicase ATP-binding domain). Contains 1 [helicase C-terminal domain](http://au.expasy.org/cgi-bin/get-similar?name=helicase C-terminal domain). Contains 1 [HSA domain](http://au.expasy.org/cgi-bin/get-similar?name=HSA domain). |
| [Q9NYI0](http://au.expasy.org/cgi-bin/get-sprot-entry?Q9NYI0)  (Swiss-Prot) | **PSD3**, EFA6R, HCA67, KIAA0942 | AML/MLL/ALL | **[FUNCTION](http://au.expasy.org/sprot/userman.html#CC_line)**: Guanine nucleotide exchange factor for ARF6-like protein.  **[SIMILARITY](http://au.expasy.org/sprot/userman.html#CC_line)**: Contains 1 [PH domain](http://au.expasy.org/cgi-bin/get-similar?name=PH domain). Contains 1 [SEC7 domain](http://au.expasy.org/cgi-bin/get-similar?name=SEC7 domain). |
| [P23511](http://au.expasy.org/cgi-bin/get-sprot-entry?P23511)  (Swiss-Prot) | **NFYA** | AML/MLL/ALL | **[FUNCTION](http://au.expasy.org/sprot/userman.html#CC_line)**: Stimulates the transcription of various genes by recognizing and binding to a CCAAT motif in promoters, for example in type 1 collagen, albumin and beta-actin genes.  **[SUBUNIT](http://au.expasy.org/sprot/userman.html#CC_line)**: Heterotrimeric transcription factor composed of three components, NF-YA, NF-YB and NF-YC. NF-YB and NF-YC must interact and dimerize for NF-YA association and DNA binding.  **[INTERACTION](http://au.expasy.org/sprot/userman.html#CCBP)**: [Q07955](http://au.expasy.org/uniprot/Q07955):SFRS1; NbExp=1; IntAct=[EBI-389739, EBI-398920](http://www.ebi.ac.uk/intact/search/do/search?binary=EBI-389739,EBI-398920);  **[SUBCELLULAR LOCATION](http://au.expasy.org/sprot/userman.html#CC_line)**: Nucleus.  **[SIMILARITY](http://au.expasy.org/sprot/userman.html#CC_line)**: Belongs to the [NFYA/HAP2 subunit family](http://au.expasy.org/cgi-bin/get-similar?name=NFYA%2FHAP2 subunit family). |
| [Q9NZL6](http://au.expasy.org/cgi-bin/get-sprot-entry?Q9NZL6)  (Swiis-Prot) | **RGL1** | AML/MLL/ALL | **[FUNCTION](http://au.expasy.org/sprot/userman.html#CC_line)**: Probable guanine nucleotide exchange factor.  **[SUBUNIT](http://au.expasy.org/sprot/userman.html#CC_line)**: Interacts with Ras [(By similarity)](http://au.expasy.org/sprot/userman.html#Non-experimental_qualifier).  **[INTERACTION](http://au.expasy.org/sprot/userman.html#CCBP)**: [P01112](http://au.expasy.org/uniprot/P01112):HRAS; NbExp=1; IntAct=[EBI-365926, EBI-350145](http://www.ebi.ac.uk/intact/search/do/search?binary=EBI-365926,EBI-350145); [Q92963](http://au.expasy.org/uniprot/Q92963):RIT1; NbExp=1; IntAct=[EBI-365926, EBI-365845](http://www.ebi.ac.uk/intact/search/do/search?binary=EBI-365926,EBI-365845); [Q99578](http://au.expasy.org/uniprot/Q99578):RIT2; NbExp=1; IntAct=[EBI-365926, EBI-365914](http://www.ebi.ac.uk/intact/search/do/search?binary=EBI-365926,EBI-365914);  **[TISSUE SPECIFICITY](http://au.expasy.org/sprot/userman.html#CC_line)**: Expressed in a wide variety of tissues with strong expression being seen in the heart, brain, kidney, spleen and testis.  **[SIMILARITY](http://au.expasy.org/sprot/userman.html#CC_line)**: Contains 1 [N-terminal Ras-GEF domain](http://au.expasy.org/cgi-bin/get-similar?name=N-terminal Ras-GEF domain). Contains 1 [Ras-associating domain](http://au.expasy.org/cgi-bin/get-similar?name=Ras-associating domain). Contains 1 [Ras-GEF domain](http://au.expasy.org/cgi-bin/get-similar?name=Ras-GEF domain). |
| [Q9UL49](http://au.expasy.org/cgi-bin/get-sprot-entry?Q9UL49)  (Swiss-Prot) | **TCFL5,** CHA, E2BP1  ` | AML/MLL/ALL | **[FUNCTION](http://au.expasy.org/sprot/userman.html#CC_line)**: Putative transcription factor. Isoform 1 may play a role in early spermatogenesis.  **[SUBUNIT](http://au.expasy.org/sprot/userman.html#CC_line)**: Efficient DNA binding requires dimerization with another bHLH protein.  **[SUBCELLULAR LOCATION](http://au.expasy.org/sprot/userman.html#CC_line)**: Nucleus.  **[TISSUE SPECIFICITY](http://au.expasy.org/sprot/userman.html#CC_line)**: Isoform 1 is testis specific. [Isoform 2](http://au.expasy.org/uniprot/Q9UL49-2) is pancreas specific.  **[DEVELOPMENTAL STAGE](http://au.expasy.org/sprot/userman.html#CC_line)**: Isoform 1 is specifically expressed in primary spermatocytes at the pachytene stage, but not those at leptonema stage. Not expressed in other testicular cells, including spermatogonia located in the basal compartment of the seminiferous tubule or spermatids.  **[DISEASE](http://au.expasy.org/sprot/userman.html#CC_line)**: Antibodies against TCFL5 are present in sera from patients with Chagas' disease, an autoimmune disease caused by Trypanosoma cruzi. Two different epitopes that mimic Trypanosoma cruzi antigens have been identified: R1 and R3 epitopes, which are recognized by T- and B-cells, respectively.  **[SIMILARITY](http://au.expasy.org/sprot/userman.html#CC_line)**: Contains 1 [basic helix-loop-helix (bHLH) domain](http://au.expasy.org/cgi-bin/get-similar?name=basic helix-loop-helix (bHLH) domain). |
| [O76024](http://au.expasy.org/cgi-bin/get-sprot-entry?O76024)  (Swiss-Prot) | **WFS1** | AML/MLL/ALL | **[SUBCELLULAR LOCATION](http://au.expasy.org/sprot/userman.html#CC_line)**: Endoplasmic reticulum; endoplasmic reticulum membrane; multi-pass membrane protein.  **[TISSUE SPECIFICITY](http://au.expasy.org/sprot/userman.html#CC_line)**: Highly expressed in heart followed by brain, placenta, lung and pancreas. Weakly expressed in liver, kidney and skeletal muscle. Also expressed in islet and beta-cell insulinoma cell line.  **[POLYMORPHISM](http://au.expasy.org/sprot/userman.html#CC_line)**: Arg-456-His, Arg-611-His and Ile-720-Val polymorphisms are in tight linkage disequilibrium with one another and associated with type 1 diabetes in Japanese.  **[DISEASE](http://au.expasy.org/sprot/userman.html#CC_line)**: Defects in WFS1 are the cause of Wolfram syndrome (WFS) [[MIM:222300](http://www.ncbi.nih.gov/entrez/dispomim.cgi?id=222300)]; also known as diabetes insipidus and mellitus with optic atrophy and deafness syndrome (DIDMOAD). It is a rare autosomal recessive disorder characterized by juvenile diabetes mellitus, diabetes insipidus, optic atrophy, deafness and various neurological symptoms. |

**REFERENCES**
